# Seroprevalence of *Toxoplasma gondii* in Cattle in Southern Egypt: Do Milk and Serum Samples Tell the Same Story?

**DOI:** 10.3390/ani14213122

**Published:** 2024-10-30

**Authors:** Ragab M. Fereig, El-Sayed El-Alfy, Azzah S. Alharbi, Mona Z. Abdelraheem, Abdulaziz M. Almuzaini, Mosaab A. Omar, Omnia M. Kandil, Caroline F. Frey

**Affiliations:** 1 Division of Internal Medicine, Department of Animal Medicine, Faculty of Veterinary Medicine, South Valley University, Qena 83523, Egypt; 2 Parasitology Department, Faculty of Veterinary Medicine, Mansoura University, Mansoura 35516, Egypt; sydnabil@mans.edu.eg; 3 Department of Clinical Microbiology and Immunology, Faculty of Medicine, King Abdulaziz University, Jeddah 21589, Saudi Arabia; asalharbi3@kau.edu.sa; 4 Special Infectious Agents Unit, King Fahd Medical Research Center, King Abdulaziz University, Jeddah 21362, Saudi Arabia; 5 The National Institute of Oceanography and Fisheries (NIOF), Aswan 81511, Egypt; monazaki89@gmail.com; 6 Department of Veterinary Preventive Medicine, College of Veterinary Medicine, Qassim University, Buraydah 51452, Saudi Arabia; ammzieny@qu.edu.sa; 7 Department of Pathology and Laboratory Diagnosis, College of Veterinary Medicine, Qassim University, Buraydah 51452, Saudi Arabia; mos.mohamed@qu.edu.sa; 8 Department of Parasitology and Animal Disease, Veterinary Research Institute, National Research Centre, Giza 12511, Egypt; kandil_om@yahoo.com; 9 Department of Infectious Diseases and Pathobiology, Institute of Parasitology, Vetsuisse-Faculty, University of Bern, Länggassstrasse 122, CH-3012 Bern, Switzerland

**Keywords:** antibodies, cattle, epidemiology, ELISA, infection, *Toxoplasma gondii*

## Abstract

*Toxoplasma gondii* is an intracellular protozoan parasite of veterinary and public health importance. Infection may lead to abortion in susceptible pregnant animals and women, and potentially fatal health complications in immunocompromised individuals. In this study, we aimed to provide an update on the seroprevalence of, and risk factors for, *T. gondii* antibodies in cattle from Qena, southern Egypt. Additionally, we investigated if raw milk and serum samples from the same animals reacted similarly in a commercial ELISA, thus potentially reducing the invasiveness of future serosurveillance studies. Cattle serum samples (*n* = 362) from three locations in the Qena governorate (Qena, Qus, and Al Waqf cities), of both sexes and different ages were collected. From most dairy cows, a corresponding milk sample (*n* = 154) was additionally obtained. We found that the overall seroprevalence in serum samples was 9.1% (33/362). Increasing age was the sole risk factor identified in our study among all tested parameters (location, age, sex, lactating yes or no). Thus, older cattle (more than 3 years old) exhibited significantly higher rates of *T. gondii* antibodies (11.7%; *p* = 0.033, odd ratio = 4.3) in comparison to animals younger than 1 year (2.9%). In the corresponding serum and milk samples, the prevalence was 12.3% (19/154) in serum samples, and 9.7% (15/154) in milk samples, respectively. A high correlation was observed between the two sample types with a concordance of 97.4%, a kappa value of 0.87, and a Pearson r correlation coefficient of 0.85. When the serum ELISA was taken as the gold standard, the milk ELISA had the following characteristics: sensitivity (78.9%), specificity (100%), positive predictive value (100%), negative predictive value (97.1%), and area under the receiver operating characteristic (ROC) curve (0.6, *p* = 0.0011). In this study, we confirmed the frequent occurrence of *T. gondii* antibodies in cattle in southern Egypt and demonstrated that non-invasive milk samples may be used instead of serum samples for seroprevalence studies in dairy cows.

## Simple Summary

The intracellular zoonotic protozoan parasite *Toxoplasma gondii* affects humans and animals worldwide. Consuming *T. gondii* infected undercooked meat, raw milk, or their byproducts poses a significant risk to humans. Therefore, it is crucial to maintain monitoring of the prevalence of *T. gondii* in food animals. Little is known about *T. gondii* prevalence in cattle in Egypt. This study was conducted in Qena, southern Egypt, and specific antibodies to *T. gondii* were identified in 9.1% (33/362) of serum samples using commercial ELISA. The only identified risk factor for increased seroprevalence was the animals’ increasing age. This survey revealed a prevalent *T. gondii* infection in cattle herds in the Qena governorate and updated information on *T. gondii* in cattle in Egypt. Additionally, 154 milk samples were taken from the sampled dairy cows and tested for *T. gondii* antibodies. There was a strong association between the serum and milk samples, with a prevalence of 12.3% (19/154) in serum samples and 9.7% (15/154) in milk samples, respectively. This suggests that the non-invasive and simple-to-obtain milk samples could be a suitable replacement for blood samples in the detection of *T. gondii* antibodies in dairy animals.

## 1. Introduction

*Toxoplasma gondii* is a widespread protozoan parasite that can infect many hosts, including humans, animals, and marine mammals [1,2,3]. The definitive hosts of the parasite are cats, which shed oocysts that are resistant to environmental conditions. Other animals, including cattle, become infected with *T. gondii* after ingesting the sporulated oocysts shed by cats [3,4]. *Toxoplasma gondii* infections in livestock can pose a potential risk to public health through ingestion of tissue cysts in raw or undercooked meat of infected animals [5]. The consumption of raw milk and milk byproducts was significantly correlated with the risk of human toxoplasmosis, particularly caprine products [5,6,7].

Globally, approximately one-third of human beings are infected with *T. gondii*, although this varies greatly among populations [3,8]. In immunocompetent individuals, the majority of infections appear to be asymptomatic. Contrastingly, in immunocompromised individuals and fetuses, the parasite can cause significant illness [9]. Abortions have been reported as the most important clinical manifestation in farm animals, especially in sheep, and can lead to economic losses. However, reports on clinical toxoplasmosis in naturally infected cattle are rare [10,11,12]. There is little evidence of the existence of viable *T. gondii* infections, even though a number of investigations employing PCR methods revealed up to 10 or 20% of *T. gondii*-positive cattle tissues [10,11]. The parasite DNA was detected in cow’s milk samples from Brazil (2.8%) [13], Iran (3.5%) [14], and Poland (15.9%) [15]. Furthermore, it was revealed that consuming raw cow’s milk byproduct was linked to *T. gondii* human infection [16]. Additionally, tachyzoites survived in the pH conditions of cow’s milk, which may suggest that unpasteurized cow’s milk might transmit infection [17].

Many reports have investigated *T. gondii* infections in humans and animals in Egypt but the current situation of toxoplasmosis in Egypt is unclear [18]. Physicians commonly believe that toxoplasmosis is the cause of abortions and pregnancy-related complications, but the published literature lacks a clear diagnosis and is poorly structured. Considerable studies in Egypt have investigated the role of *T. gondii* in congenital toxoplasmosis in humans, but they lacked a precise diagnosis, and the majority of them rely on serological findings. There have also been reports of ocular toxoplasmosis with symptoms of uveitis and chorioretinitis based on positive serology and the lesion [18]. In Egypt, toxoplasmosis-related animal abortions were only documented in sheep and goats. *Toxoplasma gondii* infection was detected by serological testing in aborted ewes and does, and *T. gondii* DNA was found in the tissues of the aborted fetuses [19]. Furthermore, Toxoplasma antibodies were detected in pregnant ewes and does from a flock with a history of abortion [20,21].

Several studies on the seroprevalence of *T. gondii*-specific antibodies in cattle have been published during the past few decades, with an estimated global seroprevalence of 16.94% [22]. There is a lack of studies investigating cattle, especially in southern Egyptian regions [18]. The cattle population in Egypt is estimated at approximately 5.1 million heads [23]. Two studies tested cattle from northern Egypt for antibodies to *T. gondii* and found seroprevalences of 10.75% and 5.3%, respectively [24]. Only one study investigated cattle in southern Egypt and found a seroprevalence of 23.6% using the latex agglutination test and TgGRA7-based ELISA [25]. The aim of this study was to (1) update the prevalence and assess the risk factors associated with *T. gondii* antibodies in cattle in Qena governorate, southern Egypt, and (2) to assess whether non-invasive milk samples can be used instead of serum samples for such surveys in dairy cows.

## 2. Materials and Methods

### 2.1. Ethical Approval

This study was carried out in compliance with the guidelines set forth by the South Valley University, Qena, Egypt, Faculty of Veterinary Medicine Research Board. It was approved by South Valley University’s Research Code of Ethics under code number 36 (RCOE-36).

### 2.2. Description of Animal and Region of the Study

**Figure 1:**
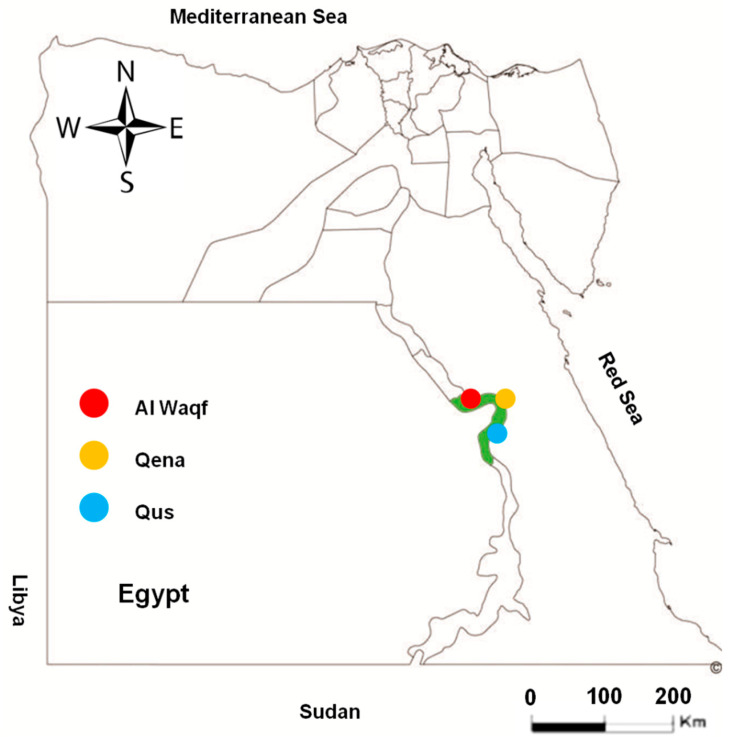
Map of Egypt showing the place of sample collection. Area with green color in the map refers to the investigated Qena governorate. Colored circles show the different cities of Qena that were investigated in this study.

A total of 362 randomly collected serum samples from apparently healthy cattle were obtained from different locations in the Qena governorate, southern Egypt (Qena, Qus, and Al Waqf cities; Figure 1). The samples represented cattle of different sexes (male and female) and various age groups. To determine the risk factors for toxoplasmosis infection in the investigated animals, we assessed the location, sex, age, and lactation. From 154 dairy cows, a milk sample was obtained in addition to the serum sample (Table 1). The availability of samples and owner cooperation determined the numbers and groups of tested animals in this study.

### 2.3. Sample Collection and Preparation

The blood samples were obtained using the jugular vein puncture procedure from the studied cattle and placed into glass tubes devoid of anticoagulant. After centrifuging the blood at 2200× *g* for 15 min at room temperature, the serum was collected and stored at −20 °C until analysis.

Milk samples were centrifuged at 1000× *g* for 10 min and lactoserum was extracted from the layer beneath the cream. The lactoserum was then kept at −20 °C until use.

### 2.4. ELISA for Antibody Detection

Anti-*T. gondii* antibodies were detected using the indirect multi-species ELISA assay for *Toxoplasma gondii* detection (ID.vet, Grabels, France) following the manufacturer’s guidelines. Briefly, both test serum samples and controls were subjected to a 1:10 dilution. Lactoserum samples were used undiluted, as outlined earlier [26,27]. The proportion of sample (S) to positive (P) ratio (S/P%) for each tested sample was calculated using the optical density (OD) values and the following formula: S/P (%) = (OD sample − OD negative control)/(OD positive control − OD negative control) × 100. A sample was regarded as negative if the S/P% was less than 40%, doubtful if S/P% was between 40% and 50%, and the test was deemed positive if the S/P% was greater than 50%. All ELISA results were measured at 450 nm using an Infinite^®^ F50/Robotic ELISA reader (Tecan Group Ltd., Männedorf, Switzerland) to determine the optical densities (ODs).

### 2.5. Statistical Analysis

The differences in the seroprevalence rates and associated risk factors were assessed with the Fisher exact probability test (two-tailed), along with 95% confidence intervals (including continuity correction), and odds ratios. This analysis was conducted using the online statistical platform www.vassarstats.net (accession on 26 September 2024). GraphPad Prism version 5 (GraphPad Software Inc., La Jolla, CA, USA) was used to estimate *p*-values. The Bonferroni post hoc test was used after one-way analysis of variance (ANOVA) to determine the significance between various ELISA groups. A *p*-value < 0.05 indicated that the difference was significant [28,29].

## 3. Results

### 3.1. Seroprevalence and Risk Factor Analysis of Toxoplasma gondii Seropositivity in Cattle in Qena

Out of 362 cattle examined, 33 serum samples tested positive for *T. gondii* antibodies (9.1%; 95% CI: 6.4–12.7) using a commercial iELISA. Among the 362 examined cattle, 154 cows had both raw milk and serum samples available for testing. Out of these, 19 serum samples and 15 milk samples tested positive for specific antibodies against *T. gondii*, resulting in seropositive rates of 12.3% (95% CI: 7.8–18.8) and 9.7% (95% CI: 5.7–15.8), respectively. It was observed that four cows had positive serum samples but negative milk samples (Table 2).

The prevalence of *T. gondii* antibodies in tested cattle from Qena, southern Egypt, was evaluated in relation to the following available variables: location, sex, and age. Only age had a significant impact on the seropositive rate among these variables. Cattle over the age of three years had a significantly higher prevalence of *T. gondii* antibodies (11.7%; odds ratio = 4.3; *p* = 0.033) than the reference group of animals younger than one year (2.9%) (Table 3). According to the lactation status of adult dairy cows ≥2.5 years old (*n* = 168), *T. gondii* antibodies were detected only in 10 (6.5%) samples collected from lactating cows.

**Figure 2:**
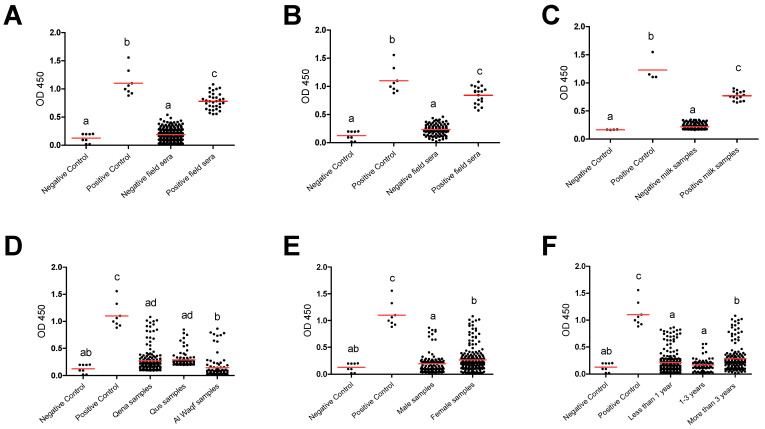
Reactivity of anti-*Toxoplasma gondii* antibodies in tested cattle groups. All samples (dots) were tested against control negative and positive samples provided by the commercial ELISA kit for detection of specific antibodies against *T. gondii* and classified as positive or negative according to the manufacturer’s instructions. (**A**) OD values of serum samples from all tested cattle (*n* = 362), (**B**) serum samples from dairy cows (*n* = 154), and (**C**) corresponding milk samples from dairy cows (*n* = 154). OD values of serum samples in relation to the different locations (**D**), sexes (**E**), and ages (**F**). Each red line represents the mean of each group. The different letters above the groups in the graphs indicate statistically significant differences in other groups (one-way ANOVA with Bonferroni post hoc analysis, *p* < 0.05).

Furthermore, group comparisons of ELISA optical densities (OD) from negative and positive controls, as well as from different groups, were conducted to evaluate the seroreactivity against *T. gondii*. In all tested groups, the OD values for the antibody levels from the positive controls were considerably greater than those from the negative samples and negative controls. Furthermore, compared to negative field samples and negative controls, positive field sera were substantially higher. This outcome demonstrates the efficacy and validity of the iELISA kits employed in our investigation to effectively separate the tested samples’ seroreactivity levels. The substantial difference between the positive and negative samples as well as the similar outcomes between the positive samples and the positive controls suggested this impact. This effect was indicated in the significant difference between positive and negative samples and the comparable results between positive samples and positive controls in totally used serum samples (*n* = 362) (Figure 2A). A similar effect was also observed when a comparison was conducted for the serum samples (Figure 2B) and milk samples (Figure 2C) collected from lactating cows (*n* = 154). Also, a comparison was conducted among negative and positive controls, as well as different factors such as location (Qena vs. Qus vs. Al Waqf cities), sex (males vs. females), and age groups (<1 year old vs. ≥1–<3 years old vs. ≥3 years old). The OD values of samples from Qena and Qus cities were markedly higher than those from Al Waqf city (*p* < 0.05) (Figure 2D). The OD values from female samples were higher than those from male samples (Figure 2E). Notably, samples belonging to older aged cattle (≥3 years old) exhibited higher OD values compared to those from both young (<1 year old) and mid-aged cattle (≥1–<3 years old) (*p* < 0.05) (Figure 2F).

### 3.2. Analysis of the Correlation Between Milk and Serum Samples Using the Same Commercial ELISA

According to Vassarstats.net, online software analysis, the estimated prevalence was 12.3% (CI 95%; 7.7–18.8). This value is consistent with the data we manually computed from the serum antibody level (Table 2). In comparison to serum antibody ELISA for cow’s milk, the results showed that the milk antibody ELISA had the following characteristics: sensitivity, specificity, positive predictive value, false positive, negative predictive value, and false negative value of 78.9%, 100%, 100%, 0%, 97.1%, and 2.9%, respectively. Moreover, when comparing milk and serum tests from the same animal and using the same ELISA assay, our testing approach showed a high concordance (97.4%) and a significant kappa value (0.87) (Table 4).

**Figure 3:**
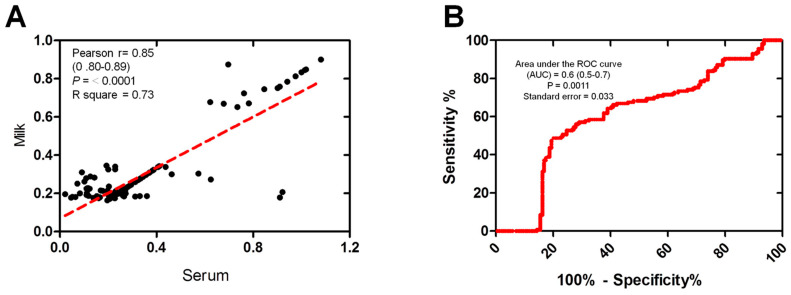
The correlation between milk and serum samples reactivity. (**A**) Correlation between milk and the corresponding serum samples using the same ELISA. Scatter graphs show the correlation between OD values recorded for milk and serum samples (*n* = 154 each) from the same animals. The equation represents the approximation formula. The break line represents the calculated line of best fit. Correlation coefficients were calculated using Pearson’s correlation coefficient: |r| = 0.70, strong correlation; |r| > 0.5–<0.7, moderately strong correlation; and |r| = 0.3–0.5 weak-to-moderate correlation. (**B**) Receiver operating characteristic (ROC) curve values were calculated using the area under the curve (AUC) as a diagnostic accuracy test to validate the milk against serum samples of the same animals (*n* = 154) using the same ELISA kit. The ROC curve for the milk samples against serum samples shows an area under the curve of 0.6 (95% CI: 0.5–0.7).

The correlation between the serum and milk antibodies OD readings, which were both measured using the same ELISA test, was also examined. Scatter plots illustrate the correlation between the OD values obtained from milk and serum samples within the tested group (*n* = 154). A strong correlation was observed between the milk and serum samples using ELISA OD (Pearson’s r = 0.85, *p* ≤ 0.0001, R square = 0.73) (Figure 3A). The accuracy of the immunoassays for ELISA was assessed using the area under the receiver operating characteristic (ROC) curve (AUC). The estimated AUC was 0.6 (CI 95%: 0.5–0.7), indicating the moderately high performance of the milk samples in comparison to the serum samples (Figure 3B).

**Figure 4:**
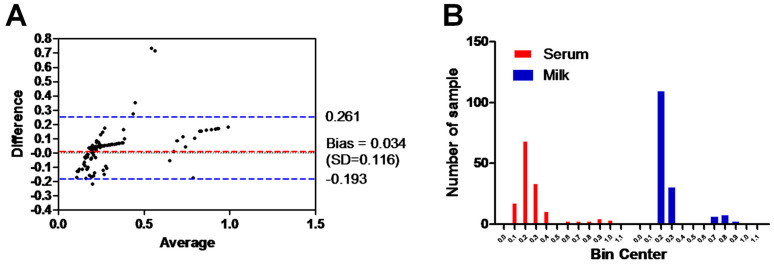
Analysis of the results between milk and serum samples. (**A**) Bland–Altman plot of ELISA testing between the milk and serum samples (shown as black dots). Dotted bluish lines between 0.2 and −0.193 of the standard deviation of 0.116 from mean (dotted red line). (**B**) Histogram of the tested milk samples against serum samples using the same ELISA shows the frequency distribution of obtained data. The x axis represents the OD values and the y axis indicates the number of samples represented in each bar. The frequency distribution of obtained data using serum milk samples was 0.27 ± 0.17 SD and using serum samples was 0.31 ± 0.22 SD.

The Bland–Altman plot of ELISA testing between the milk and serum samples of the same animals and using the same ELISA test was performed. The range of the dotted blue lines is 0.261 to −0.193 of the standard deviation (SD), which is 0.034 from the mean (dotted red line). The majority of data points indicate good agreement between the two samples, falling within the range of ±0.116 SDs (Figure 4A). Additionally, the histogram of the milk and serum samples tested with the same ELISA assay demonstrated a strong correlation in the frequency distribution of the obtained data (milk samples, 0.27 ± 0.17 SD; serum samples, 0.31 ± 0.22 SD) for the total number of values (*n* = 154). Majority of samples laid between OD value 0.2 and 0.3 for milk (90.3%; 139/154) and serum samples (66.9%; 103/154) (Figure 4B).

## 4. Discussion

In this study, we conducted a cross-sectional study to update the prevalence and the risk factors associated with *T. gondii* antibodies in cattle in Qena governorate, southern Egypt, and to analyze the correlation between the antibody reactivity in milk and serum samples. There are various serological tests available to detect *T. gondii* infection, of which the ELISA has been the most commonly used method [9,30,31]. When testing samples, using milk instead of serum offers a range of benefits. Not only is collecting milk samples simpler and more cost-effective, but it also reduces the risk of unintentional needle-transmitted diseases and minimizes the productivity losses associated with stress [32]. Even if parasites cannot be directly detected, seroprevalence can indicate the risk of human infection from raw milk consumption if there is a correlation between the presence of viable parasites and the detection of antibodies to *T. gondii*.

Herein, the total seroprevalence of *T. gondii* using serum samples from all tested cattle (*n* = 362) was 9.1%. This seropositive rate was lower than that reported in our previous study conducted using cattle (24.4%) from Qena and (21.1%) from Sohag, Egypt [25]. This discrepancy might be related to using a different ELISA approach based on *T. gondii* dense granule protein 7 (TgGRA7) antigen in the previous study, added to the difference in time, place, and animals of sample collection. However, our seropositive rate was similar to that reported in cattle (10.75%) from Sharkia, northern Egypt, using *T. gondii* surface antigen 2 (TgSAG2)-based ELISA [24] and higher than that detected in cattle (5.3%) from Beheira, northern Egypt, using the same commercial ELISA kit [33]. Recent meta-analysis studies on a worldwide scale predicted pooled prevalences that were similar to the seroprevalence found in the cattle under investigation. For cattle globally, a pooled seroprevalence of 16.94% was calculated [22]. The prevalence of *T. gondii* infection in 3366 cattle studied in Africa varied from 3.6% to 32%, with an overall estimated frequency of 12% [34]. The total pooled *T. gondii* seroprevalence in cattle in China was 10.1% (4217/39,274), which is comparable to our prevalence [35]. Although country-specific seroprevalence varies greatly, a higher overall pooled prevalence of *T. gondii* in cattle was found at 31% when compared to the neighboring country, Sudan [36].

In the 154 cows for which raw milk samples and serum samples were available, 19 serum samples and 15 milk samples tested positive, resulting in seropositive rates of 12.3% and 9.7%, respectively. Therefore, four cows had milk samples testing negative despite having positive serum samples. The stage of lactation can affect the antibody levels in both serum and milk samples [37]. However, the natural levels of immunoglobulins in cow’s milk during lactation may cause varying prevalence in serum and milk samples, requiring further investigation. Our detected rate (9.7%) was higher than that detected by our previous report conducted using cattle (2.4%) from the Sohag governorate and using the same ELISA [12]. This latter was a pioneering study that provided valuable and novel data on serological and molecular detection of *T. gondii* in individual and bulk samples of raw milk of different ruminant animals from different Egyptian regions. However, the unavailability of corresponding serum samples was considered a limitation that we attempted to avoid in the present study. Thus, the data of the current study could be regarded as a confirmatory record regarding the usefulness of commercial ELISA and protocols in the detection of anti-*T. gondii* antibodies in bovine milk.

A risk factor assessment was conducted based on serum testing of the total cattle population. In the current study, we analyzed location, age, sex, and lactation as risk factors for *T. gondii* seropositivity. However, only age was identified as a risk factor for infection because the older aged cattle had a significantly higher seroprevalence than young cattle. The effect of location and sex was similar to that reported previously in cattle from Qena and Sohag by our group [25], but it conflicted with the effect of age because no difference was detected according to age. Globally, age was considered as an important risk factor for *T. gondii* seropositivity when cattle were investigated in Switzerland [38], and in Portugal [39]. Furthermore, Klun et al. (2006) [40] detected the location but not the sex or age as a risk factor for *T. gondii* infection in cattle from Serbia. Age but not sex also influenced the seropositivity of *T. gondii* in cattle from China [41].

In addition to our previous data [27], the findings of the current study corroborated the suitability of milk samples from cattle in monitoring *T. gondii* antibodies. This was supported by the good agreement between the results of the milk samples when tested against serum samples of the corresponding cows and using the same ELISA kit. These results were analyzed using quantitative (number of positive vs. negative samples) and qualitative (OD values of positive vs. negative samples) approaches. Several statistical analysis tests were used, indicating the utility of milk samples for the detection of *T. gondii* antibody using our employed commercial ELISA. Online software analysis (Vassarstats.net) demonstrated high sensitivity, specificity, PPV, and NPV. Also, the Pearson r correlation coefficient, the area under the ROC curve, the Bland–Altman plot, and the histogram of ELISA revealed a high correlation between analyzed milk and bovine serum. These parameters are commonly used in analyzing different diagnostic approaches either for methods or samples and great adequacy has been reported [42,43,44,45]. However, further studies testing combined milk and serum samples from cattle and other animal species are required to further validate such results.

## 5. Conclusions

In this study, we present an update on the seroprevalence of *T. gondii* antibodies in cattle from Qena, southern Egypt. Increasing age of the animals was identified as the sole risk factor for higher seroprevalence in our study. Corresponding serum and milk samples of dairy cows were highly correlated, indicating that non-invasive and easy-to-obtain milk samples might be a valid substitute for serum samples in future studies on *T. gondii* seroprevalence in dairy animals. Our findings might be used as a platform for efficient diagnosis of *T. gondii* in Egypt and consequently assist in the development of more potent control strategies for such infection.

## Figures and Tables

**Table 1:** Details of collected samples.

Item		Serum	Milk
City	Qena	182	123
	Qus	91	18
	Al Waqf	89	13
Sex	Male	110	-
	Female	252	154
Age	Less than 1 year old	68	-
	1–3 years old	88	4
	More than 3 years old	206	150
Total		362	154

**Table 2:** Seroprevalence of *Toxoplasma gondii* antibodies in cattle of Qena, southern Egypt.

Type of Sample	No. of Tested	No. of Negative (%)	No. of Doubtful (%)	No. of Positive (%)	95% CI
Serum	362	325 (89.8)	4 (1.1)	33 (9.1%)	6.4–12.7
Milk	154	139 (90.3)	0 (0)	15 (9.7)	5.7–15.8
Both	154	135 (87.7)	0 (0)	19 (12.3)	7.8–18.8

95% CI calculated according to method described by (http://vassarstats.net/, access on 26 September 2024).

**Table 3:** Risk factors for *Toxoplasma gondii* antibodies in cattle of Qena, southern Egypt.

Variables	No. of Tested	No. of Negative (%)	No. of Positive (%)	OR (95% CI) ^#^	*p*-Value *
**Place**					
Qena	182	162 (89)	20 (11)	2.1 (0.8–5.9)	0.182
Qus	91	86 (94.5)	5 (5.5)	Ref	Ref
Al Waqf	89	81 (91)	8 (9)	1.7 (0.5–5.4)	0.402
**Sex**					
Male	110	101 (91.8)	9 (8.2)	Ref	Ref
Female	252	228 (90.5)	24 (9.5)	1.2 (0.5–2.6)	0.843
**Age**					
Less than 1 year old	68	66 (97.1)	2 (2.9)	Ref	Ref
1–3 years old	88	81 (92.1)	7 (7.9)	2.8 (0.6–14.2)	0.301
More than 3 years old	206	182 (88.3)	24 (11.7)	4.3 (1–18.9)	0.033
**Lactation**					
Yes	154	144 (93.5)	10 (6.5)	2.1 (0.1–37.9)	1.000
No	14	14 (100)	0 (0)	Ref	Ref

^#^ Odds ratio at 95% confidence interval as calculated by http://vassarstats.net/ (accessed on 26 September 2024). * *p* value was evaluated by Fisher’s exact probability test (two-tailed). Ref.; value used as a reference.

**Table 4:** Diagnostic parameters of serum compared to raw milk samples in tested cows using ELISA.

Parameter	Estimated Value	95% Confidence Interval	
		Lower Limit	Upper Limit
Estimated prevalence	12.3	7.7	18.8
Sensitivity (%)	78.9	53.4	93.0
Specificity (%)	100	96.5	100
Positive Predictive Value (%)	100	74.6	100
False positive	0	0	25.3
Negative Predictive Value (%)	97.1	92.3	99.1
False negative	2.9	0.9	7.7
Concordance (%)	97.4	93.1	99.2
Kappa value	0.87	0.74	0.99

The diagnostic parameters were analyzed using an online statistical website www.vassarstats.net, accessed on 26 September 2024. The strength of agreement was graded with kappa values of fair (0.21–0.40), moderate (0.41–0.60), and substantial (0.61–0.80).

## Data Availability

All data generated and analyzed during this study are included in this published article. Raw data supporting the findings of this study are available from the corresponding author on request.
